# Identification of KIF21B as a Biomarker for Colorectal Cancer and Associated with Poor Prognosis

**DOI:** 10.1155/2022/7905787

**Published:** 2022-11-21

**Authors:** Shanshan Xu, Youran Li, Hua Huang, Xian Miao, Yunfei Gu

**Affiliations:** ^1^Major of Chinese Medicine Surgery, Nanjing University of Chinese Medicine, Nanjing, Jiangsu 210000, China; ^2^Department of Colorectal Surgery, Affiliated Hospital of Nanjing University of Chinese Medicine, Nanjing, Jiangsu 210000, China; ^3^Department of Anorectal, Changshu Hospital Affiliated to Nanjing University of Chinese Medicine, Nanjing, Jiangsu 215500, China; ^4^Department of Oncology, Nantong Hospital of Traditional Chinese, Nantong, Jiangsu 226001, China

## Abstract

**Objective:**

This study is aimed at exploring the function of KIF21B in colorectal cancer.

**Methods:**

The expression of KIF21B was analyzed by the UALCAN database, GEPIA site, and TIMER site. The survival rate was analyzed by Kaplan-Meier curves, and the prognosis was analyzed by ROC. Relevant signaling pathways and biological processes were analyzed by GO-KEGG enrichment analysis. The correlation between KIF21B and cancer immune infiltrates was analyzed by TIMER. The functional state of KIF21B in various types of cancers was conducted by single-cell RNA-sequencing. Furthermore, the expression of KIF21B was verified by real-time qPCR and Western blotting. The cell proliferation was measured by CCK8 assay. The cell apoptosis was analyzed by flow cytometry. Cell migration and invasion were determined by the transwell assay.

**Results:**

Combination analysis of bioinformatics methods revealed that KIF21B is high expression in CRC, associated with poor survival. KIF21B and associated genes were significantly enriched in covalent chromatin modification. The expression of KIF21B was positively correlated with infiltrating levels of CD4+ T cells and neutrophils, cell apoptosis, and metastasis. KIF21B was upregulated expression in CRC cell lines. KIF21B deficiency reduced cell proliferation, migration, and invasion.

**Conclusions:**

Our study suggested that KIF21B may be a biomarker in CRC.

## 1. Introduction

Colorectal cancer is the third most common cancer in the world and the second leading cause of cancer-related deaths. In 2018, there were approximately 1.8 million new cases and 860,000 deaths [1]. By 2040, the annual global burden of colorectal cancer is expected to increase to more than 3 million new cases and 6 million deaths [1]. The incidence of colorectal cancer varies between countries, and studies on international immigrants have shown that diet and other lifestyle factors play a role in disease progression [2]. Therefore, people are paying more and more attention to formulating public health programs to reduce the incidence of colorectal cancer by targeting modifiable risk factors. Despite advances in treatment and early diagnosis in recent decades, the 5-year survival rate of CRC patients is still unsatisfactory [3]. At present, the prognosis model established based on clinical predictive indicators such as age, gender, and TNM staging is a commonly used clinical prognosis model for CRC. However, due to the high degree of heterogeneity of the disease, the prognosis based on conventional clinical predictors is not accurate, resulting in inaccurate prediction of the survival of CRC patients [4]. Therefore, the establishment of more comprehensive predictive indicators has important implications for more effective treatment.

Kinesin superfamily proteins belong to a class of microtubule-dependent molecular motors that utilize ATP hydrolysis yield in eukaryotes to move cargo such as proteins, macromolecules, and organelles such as chromosomes and vesicles along the cytoskeletal microtubule network. They play important roles in all aspects of intracellular trafficking and are involved in a wide variety of physiological processes, including embryonic development, axonal transport, and cell division. Many kinesins play important roles in cell division, and kinesin overexpression is associated with cancers such as retinoblastoma [5]. Recent study reported that KIFs participate in the division of mitotic cells by participating in the movement of chromosomes and spindles, suggesting that the release of KIFs may be related to the occurrence of tumors [6]. KIF21B is a classical kinesin that inhibits the growth of microtubules through the tail domain and participates in the regulation of microtubule dynamics as a potential microtubule suspension factor [7, 8]. Both play important roles in intercellular signal transduction, malignancy, tumorigenesis, and metastasis [9]. Studies have shown that increasing expression of KIF21B is correlated with poor disease-free survival in patients with prostate cancer [10]. KIF21B is upregulated in hepatocellular carcinoma and is significantly associated with prognosis [11]. KIF21B is abnormally expressed in osteosarcoma and affects the proliferation and apoptosis of osteosarcoma cells by regulating the PI3K/AKT pathway [12]. However, the function of KIF21B in colorectal cancer has not been reported.

In this study, the TCGA data platform was used to analyze the expression of KIF21B in colorectal cancer and its impact on survival and prognosis and to provide potential molecular markers for the diagnosis and treatment of colorectal cancer.

## 2. Methods and Materials

### 2.1. Cells and shRNA

NCM460, HT29, HCT116, SW48, and HCT15 cells were purchase from Procell. All cells were cultured in DMEM with 10% FBS and penicillin-streptomycin. shRNA were purchased from Sigma. Lipofetamine 3000 reagent was used to related transfection assays as direction of manual handbook.

### 2.2. Gene Expression Analysis

The expression of KIF21B in COAD was analyzed by online platform, including TIMER (https://cistrome.shinyapps.io/timer/), UALCAN (http://ualcan.path.uab.edu/analysis.html), and GEPIA (http://gepia.cancer-pku.cn/).

### 2.3. Kaplan-Meier Analysis and ROC Analysis

Based on TCGA-COAD dataset to draw the survival prognosis curve by using the survivor package in R. Based on TCGA-GTEx-COAD and TCGA-COAD dataset to do ROC analysis by using the pROC package.

### 2.4. GO-KEGG Enrichment Analysis

Firstly, the genes with an expression correlation to KIF21B in COAD were selected from UALCAN dataset, then the genes with Pearson score ≥ 0.4 were screened for GO-KEGG enrichment analysis through the enrichplot package of R.

### 2.5. Correlation Analysis

Correlation analysis was performed as described as previously depicted [13, 14]. The correlation between KIF21B and genes associated with immune infiltration were analyzed by Spearman score. The data comes from TIMER.

### 2.6. Single-Cell RNA-Sequence Analysis

Single-cell RNA isolation and sequencing were performed as previously depicted [15]. The functional status of KIF21B in different kinds of cancers was analyzed by CancerSEA (http://biocc.hrbmu.edu.cn/CancerSEA/). The expression profile of KIF21B in single cells obtained from CRC tissue was analyzed by t-SNE. The correlation between KIF21B and apoptosis, metastasis, DNA damage, and hypoxia was analyzed by Pearson score.

### 2.7. RT-qPCR and Western Blotting Analysis

RNA isolation and protein extraction were performed as previously described [16]. Cell were harvested, washed, and treated with TRIzol reagent for the extraction of RNA, then cDNA was produced by the reverse transcription of RNA. The primers were list as follows:

KIF21B forward: 5′-ACCTATGACTTTGTCTTCGACCT-3′;

KIF21B reverse: 5′-CAGCACCGTGGCATTATAGC-3′;

GAPDH forward: 5′-AGGTCGGTGTGAACGGATTTG-3′; and

GAPDH reverse: 5′-GGGGTCGTTGATGGCAACA-3′.

For Western blotting, protein lysate was deprived from cell lysis by RIPA buffer with protein inhibitor. And immunoblotted with the following antibodies: antimouse KIF21B (1 : 1000, Santa, sc-517174, USA) and antimouse *β*-actin (1 : 1000, Santa, sc-8432, USA).

### 2.8. Cell Viability and Apoptosis Analysis

The survival analysis of CRC cell lines was performed as described as previously depicted [17]. CCK8 assay was used to analyze cell viability. Cell lines were counted, seeded into 96-well plate, then analyzed the cell viability at 0 h, 24 h, 48 h, 72 h, and 96 h. Cell apoptosis was analyzed by flow cytometry. Cells were harvested, washed, then stained with PI or/and annexin V.

### 2.9. Cell Migration and Invasion

For migration assays, transwells with an 8 *μ*m pore size filter are inserted into a 24-well plate. The cell serum was starved overnight and then added to the upper chamber (2.5 × 10^4^ cells per insertion), and the lower chamber used a complete culture medium supplemented with 10% fetal bovine serum as a chemical attractant. After 24 hours of incubation, the remaining uninvaded cells on the upper surface of the filter were removed. The cells that passed through the filter and attached to the bottom of the membrane were fixed and stained. In each experiment, under a phase-contrast microscope, 7 areas were randomly selected from 3 repeated cells, and the number of invading cells was counted. For invasion assays, the Matrigel was spread in filter, then 4 × 10^4^ cells were seeded into chamber. And the subsequent steps were same with migration assays.

### 2.10. Statistical Analysis

Student's *t*-test and two-way ANOVA were used in statistical analyses and performed by SPSS 22.0 software. Data were presented as mean ± SEM of three independent experiments. A *P* value of 0.05 or less were considered to be significant.

## 3. Results

### 3.1. KIF21B Was Increased Expression in Colorectal Cancer

**Figure 1:**
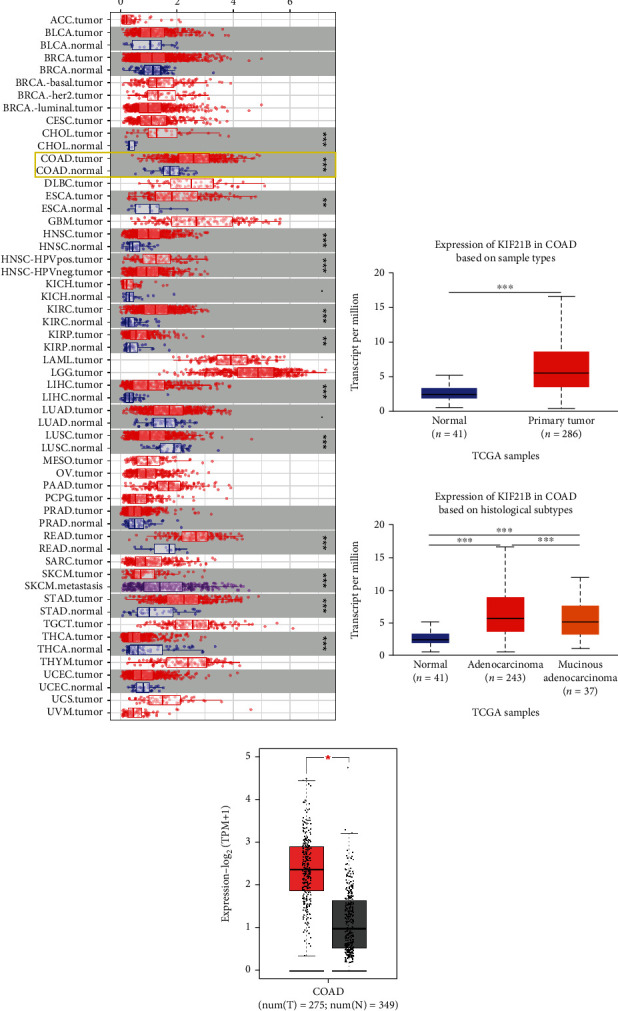
The expression of KIF21B was increased in colorectal cancer. (a) KIF21B expression in CRC was analyzed by TIMER database (https://cistrome.shinyapps.io/timer/). (b) KIF21B expression in CRC was analyzed by UALCAN database (http://ualcan.path.uab.edu/analysis.html). (c) KIF21B expression in CRC was analyzed by GEPIA database (http://gepia.cancer-pku.cn/).

First of all, we analyzed the expression of KIF21B in CRC from TIMER database. As shown in Figure 1(a), KIF21B was upregulated expression in tumor tissues compared to normal tissues of COAD. Moreover, KIF21B was also significant overexpression in primary tumor specimens compared with normal group based on the sample type in COAD. Based on the histological subtype of COAD, the expression of KIF21B was also increased expression in adenocarcinoma (*n* = 243)/mucinous adenocarcinoma (*n* = 37) compared to normal tissues (*n* = 43) from UALCAN database. In addition, KIF21B was elevated expression in tumor specimens (*n* = 275) compared with normal specimens (*n* = 349) from GEPIA database. Collectively, our data indicated that KIF21B is upregulated expression in CRC.

### 3.2. Overexpression of KIF21B in Colorectal Cancer Was Related to Poor Prognosis

**Figure 2:**
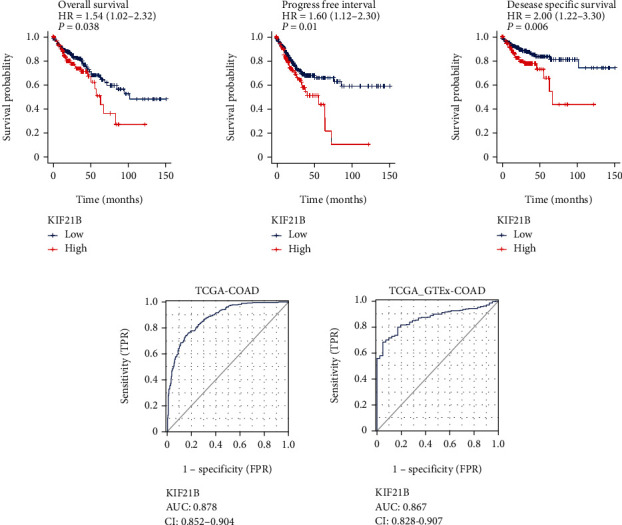
The high expression of KIF21B in colorectal cancer was related to poor prognosis. (a) Kaplan-Meier analysis of the correlation between KIF21B and prognosis from TCGA-COAD database. (b) ROC curve analysis of KIF21B in clinical diagnosis from GTEx-COAD, TCGA-COAD.

Then, we analyzed the correlation between KIF21B expression and survival of patients. As shown in Figure 2(a), there was a better survival, including overall survival, disease specific survival, and progress free interval, in patients with low expression of KIF21B compared to the high expression groups from TCGA–COAD dataset. In addition, the AUC values were all greater than 0.8, and the confidence interval was between 0.8 and 0.9 by AUC analysis from GTEx-COAD and TCGA-COAD database (Figure 2(b)). Taken together, our data suggested that KIF21B has a value as a diagnostic marker in CRC.

### 3.3. Enrichment Analysis of KIF21B Expression-Related Genes in Colorectal Cancer

**Figure 3:**
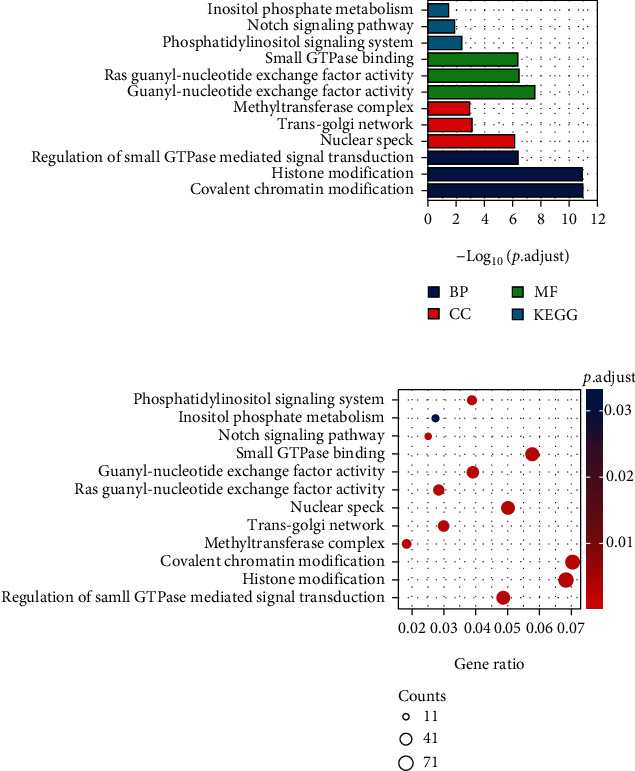
Enrichment analysis of KIF21B expression-related genes in colorectal cancer. (a, b) Based on UALCAN database analysis of genes associated with KIF21B expression in COAD, genes with Pearson CC ≥ 0.4 were selected for GO-KEGG enrichment analysis. Data was shown as column chart (a) and bubble chart (b).

**Figure 4:**
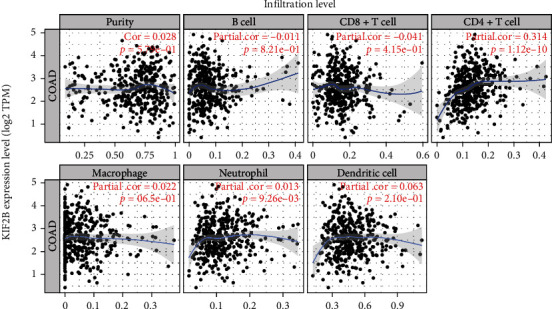
The correlation between KIF21B and immune infiltration. Correlation between KIF21B and immune infiltration in related cell lines.

In order to clarify the underlying function of KIF21B, we used GO-KEGG signal pathway analysis to confirm the networks of KIF21B in CRC. As shown in Figures 3(a) and 3(b), KIF21B mainly exerted important role in notch signaling pathway, inositol phosphate metabolism, phosphatidylinositol signaling system, and histone modification. Moreover, KIF21B and related genes are significantly enriched in covalent chromatin modification. There was a positive correlation between KIF21B expression with CD4+ T cells (cor = 0.352, *P* = 1.12e − 10) and neutrophil cells (cor = 0.352, *P* = 9.26e − 3) (Figure 4). Collectively, our data indicated that KIF21B is correlated with immune infiltrates in CRC.

### 3.4. The Correlation between KIF21B and the Biological Function of Colorectal Cancer Cells

**Figure 5:**
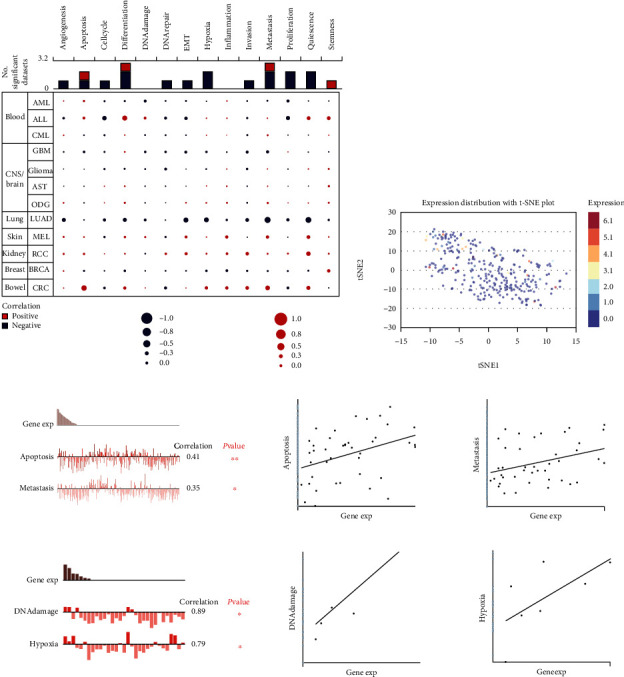
Single-cell-sequencing to analyze the correlation between KIF21B and the biological function of colorectal cancer cells. (a) Function analysis of KIF21B in different kinds of cancer CancerSEA (http://biocc.hrbmu.edu.cn/CancerSEA/). (b) The expression profile of KIF21B in single cell from CRC tissue was analyzed by T-SNE. (c) The correlation analysis of KIF21B with apoptosis and migration. (d) The correlation analysis of KIF21B with DNA damage and hypoxia.

Next, to better understand the expression correlation and underlying mechanisms of KIF21B in cancer, we analyzed the functional status of KIF21B in diversity cancers from the CancerSEA database. KIF21B has been studied at the single-cell level in 12 kinds of cancer (Figure 5(a)), including AML, ALL, CML, GBM, Glioma, AST, ODG, LUAD, MEL, RCC, BRCA, and PC. KIF21B was positively correlated with CRC cell apoptosis (cor = 0.414, *P* = 0.007) and migration (cor = 0.352, *P* = 0.024). However, KIF21B was not significantly associated with any of the 14 functional states in CRC except for apoptosis and metastasis. We also analyzed the single-cell expression distribution of KIF21B in CRC tissues by t-SNE plot, as shown in Figure 5(b), there was a differential expression of KIF21B in single-cell level. Furthermore, KIF21B expression was significantly positive correlation with apoptosis (cor = 0.41) and metastasis (cor = 0.35) (Figure 5(c)). And there was also a positive correlation between KIF21B expression and DNA damage (cor = 0.89) and hypoxia (cor = 0.79) (Figure 5(d)). Taken together, our data suggested that KIF21B is involved in the phenotype of CRC.

### 3.5. KIF21B Was Upregulated Expression in CRC Cells and Promotes Cell Migration and Invasion

**Figure 6:**
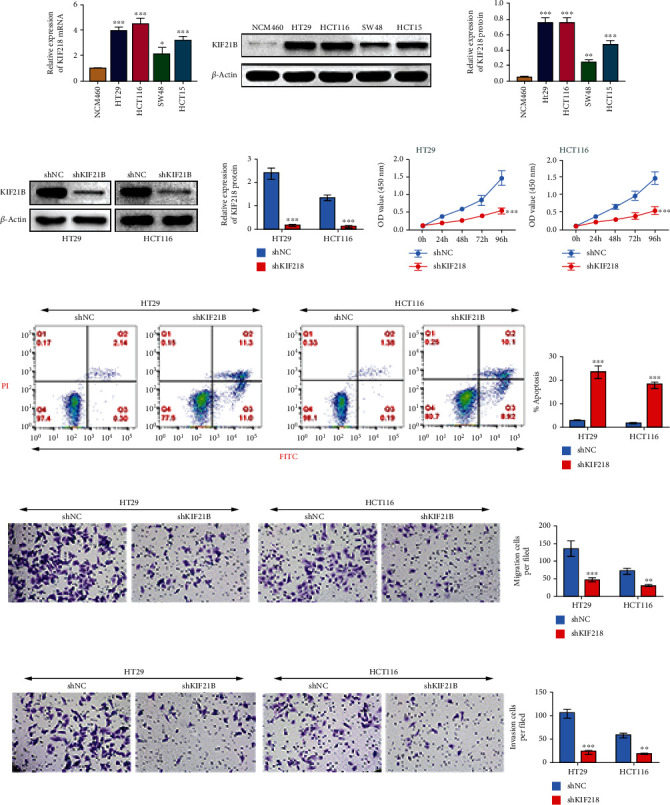
KIF21B was upregulated in colorectal cancer cells and promotes survival, increases migration and invasion. (a) KIF21B mRNA expression in CRC cell lines was measured by RT-qPCR. (b) Western blotting assay was used to analyze KIF21B protein expression in CRC cell lines. (c) HT29 and HCT116 cell lines were transfected with shNC and shKIF21B, respectively. The expression of KIF21B was measured by Western blotting. (d) KIF21B-knockdown HCT116 and HT29 cell lines were cultured as the indicated time (0 h, 24 h, 48 h, 72 h, and 96 h), then the cell proliferation was measured by CCK8 assay. (e) Cell apoptosis in KIF21B deficiency HCT116 and HT29 cell lines was measured by flow cytometry. (f, g) Cell migration and invasion were measured by transwell assay without or with matrigel.

To better clarify the function of KIF21B in CRC, we analyzed the expression of KIF21B in cell level, as shown in Figures 6(a) and 6(b). KIF21B was upregulated expression in CRC cell lines, including HT29, HCT116, SW480, and HCT15, compared with normal intestinal epithelial cells NCM460. Then, we constructed KIF21B knockdown HCT116 and HT29 cell lines by shRNA (Figure 6(c)). And we analyzed cell proliferation in the cell lines by CCK8 assay, as shown in Figure 6(d), knockdown of KIF21B decreased cell proliferation in HT29 and HCT116 cell lines. Moreover, KIF21B deficiency induced cell apoptosis in HT29 and HCT116 cell lines (Figure 6(e)). Apart from this, cell migration and invasion were also suppressed in HT29 and HCT116 cell lines with shKIF21B treatment (Figures 6(f) and 6(g)). Collectively, our data suggested that KIF21B positively regulates cell proliferation, migration, and invasion in CRC.

## 4. Discussion

Colorectal cancer (CRC) as the third most common malignancy and the second mortality in diversities of cancer, which accounts for 10.2% morbidity and 9.2% mortality in all types of cancer worldwide [1]. And with the development of society, the morbidity and mortality of CRC have increased year by year in the past three decades. Therefore, it is essential to find out the key biomarker of poor survival in regulating of CRC development and progression. In this study, KIF21B was high expression in CRC cells and tissues. Overexpression of KIF21B was associated with poor survival. KIF21B was correlated with immune infiltrates in CRC. Furthermore, single-cell sequencing indicated that KIF21B exerts positive correlation with cell apoptosis and metastasis. In addition, knockdown of KIF21B reduced cell viability, metastasis, and invasion, whereas increased cell apoptosis in CRC cell lines.

KIF21B belongs to a superfamily of motor proteins, exerted critical role in intracellular trafficking, cell mitosis, and cytoskeletal reorganization [8]. In the past few years, many research reported that the change of kinesins acts important role in cell growth, cell migration, cell invasion, and tumorigenesis in diversity of cancers, including prostate cancer, breast cancer, bladder cancer, pancreatic cancer, gastric cancer, hepatocellular carcinoma, colorectal cancer, and lung cancer [18]. And KIF21B, as a member of kinesins family proteins, also exerted important role in gastric cancer [19], HCC [11], and NSCLC [20]. Here, we were firstly demonstrated that KIF21B plays key role in CRC. Generally, KIF21B exerted a function of ATP-dependent microtubule motor protein, which participates in regulation of microtubule dynamics, including growth rate and mutation frequency [7]. Based on the important role of kinesins and microtubules in signaling transduction, transport, metastasis, malignancy, and tumorigenesis, we also clarified the key role of KIF21B in cell proliferation, apoptosis, migration, and invasion in CRC.

Previous study reported that KIF21B is overexpression in HCC cells and tissues, and KIF21B deficiency significantly suppressed cell proliferation and increased cell apoptosis, which indicated that KIF21B is a potential diagnostic and prognostic marker for HCC [11]. Moreover, Sun et al. also reported that KIF21B was overexpression in non-small-cell lung cancer tissues and associated with poor prognosis. Knockdown of KIF21B remarkably reduced cell proliferation, cell migration, cell invasion, and increased cell apoptosis in NSCLC [20]. Similarly, we confirmed the result of KIF21B in regulation of cell viability and apoptosis in CRC. Apart from this, KIF21B deficiency reduced the expression of Bcl-2 and induced the expression of Bax and active caspase 3 in NSCLC [20]. KIF21B decreasing expression facilitated cell apoptosis and impeded cell growth and tumorigenesis in nude mice through the inhibition of PI3K/AKT pathway and decreasing of Bcl-2 and increasing of Bax expression in osteosarcoma [12]. The underlying mechanism of KIF21B in regulating of cell apoptosis in CRC needs to be further investigated.

In our research, KIF21B mainly exerted important role in notch signaling pathway, inositol phosphate metabolism, and phosphatidylinositol signaling system by KEGG enrichment analysis, which was similar with the previous study of critical role of KIF21B in osteosarcoma [12]. They also demonstrated that KIF21B expression regulates cell proliferation and apoptosis through the PI3K/AKT pathway. Here, the underlying mechanism of KIF21B involved in phosphatidylinositol signaling needs further study. KIF21B was associated with DNA damage and hypoxia by single-cell sequencing. For the development and progression of cancer, hypoxia and DNA damage acted as an inducer in promoting cancer [21, 22]. Therefore, we speculated that KIF21B may induce CRC via DNA damage and hypoxia-related signal pathway by single-cell-sequencing analysis. Additionally, KIF21B was positively correlated with CD4+ T cell and neutrophil cell, which involved in regulation of immune infiltrate of cancer cells. Next, we plan to further study the underlying molecular mechanisms of KIF21B in regulation of DNA damage and immunity in CRC.

In conclusion, KIF21B was an increasing expression in Colorectal cancer cell lines and tissue specimens, which was correlated with poor survival, immune infiltrates, cell growth, and metastasis. KIF21B may be a biomarker in the diagnosis of CRC.

## Data Availability

All data generated or analyzed during this study are included in this published article.
